# Establishment and application of a CRISPR-Cas12a-based RPA-LFS and fluorescence for the detection of *Trichomonas vaginalis*

**DOI:** 10.1186/s13071-022-05475-5

**Published:** 2022-09-30

**Authors:** Shan Li, Xiaocen Wang, Yanhui Yu, Songgao Cao, Juan Liu, Panpan Zhao, Jianhua Li, Xichen Zhang, Xin Li, Nan Zhang, Min Sun, Lili Cao, Pengtao Gong

**Affiliations:** 1 State Key Laboratory for Zoonotic Diseases, College of Veterinary Medicine, Jilin University, Changchun, 130062 China; 2 Clinical Laboratory, The Second Hospital of Jilin University, Changchun, 130000 China; 3 Pingdu People’s Hospital, Qingdao, 266700 China; 4 Jilin Academy of Animal Husbandry and Veterinary Medicine, Changchun, 130062 China

**Keywords:** *Trichomonas vaginalis*, CRISPR-Cas12a, RPA, Visualization detection, On-site testing

## Abstract

**Background:**

Infection with *Trichomonas vaginalis* can lead to cervicitis, urethritis, pelvic inflammatory disease, prostatitis and perinatal complications and increased risk of HIV transmission. Here, we used an RPA-based CRISPR-Cas12a assay system in combination with a lateral flow strip (LFS) (referred to as RPA-CRISPR-Cas12a) to establish a highly sensitive and field-ready assay and evaluated its ability to detect clinical samples.

**Methods:**

We developed a one-pot CRISPR-Cas12a combined with RPA-based field detection technology for *T. vaginalis*, chose *actin* as the target gene to design crRNA and designed RPA primers based on the crRNA binding site. The specificity of the method was demonstrated by detecting genomes from nine pathogens. To improve the usability and visualize the RPA-CRISPR-Cas12a assay results, both fluorescence detection and LFS readouts were devised.

**Results:**

The RPA-CRISPR-Cas12a assay platform was completed within 60 min and had a maximum detection limit of 1 copy/µl and no cross-reactivity with *Candida albicans*, *Mycoplasma hominis*, *Neisseria gonorrhoeae*, *Escherichia coli*, *Cryptosporidium parvum*, *G. duodenalis* or *Toxoplasma gondii* after specificity validation. Thirty human vaginal secretions were tested by RPA-CRISPR-Cas12a assays, and the results were read by a fluorescent reporter and LFS biosensors and then compared to the results from nested PCR detection of these samples. Both RPA-CRISPR-Cas12a assays showed 26.7% (8/30) *T. vaginalis*-positive samples and a consistency of 100% (8/8). The RPA-CRISPR-Cas12a assays had a higher sensitivity than nested PCR (only seven *T. vaginalis*-positive samples were detected).

**Conclusions:**

The *T. vaginalis* RPA-CRISPR-Cas12a assay platform in this study can be used for large-scale field testing and on-site tests without the need for trained technicians or costly ancillary equipment.

**Graphical abstract:**

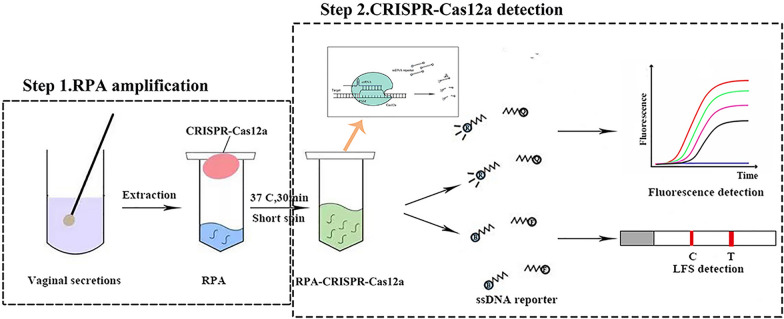

**Supplementary Information:**

The online version contains supplementary material available at 10.1186/s13071-022-05475-5.

## Background

*Trichomonas vaginalis* (TV) belongs to a group of unicellular protozoan parasites that are mainly found in the vagina and urethra [1] and is the most common sexually transmitted infection (STI) [2] worldwide [3, 4], with approximately 270 million [1] people infected each year. *Trichomonas vaginalis* infection causes inflammatory infections, such as vaginitis [3], urethritis, pelvic inflammatory disease and prostatitis [5], and can also cause perinatal complications, such as premature rupture of membranes [6] and preterm birth [7]. Meanwhile, *T. vaginalis* infection can increase the risk of HIV infection [8], cervical intraepithelial neoplasia [9] and posthysterectomy infection [10]. In some women, trichomoniasis can lead to symptoms such as vaginal erythema, production of gray-green or yellow-green secretions and difficulty in urination [11], but most infected individuals, especially men, are asymptomatic [12]. Therefore, accurate diagnosis is crucial for the proper treatment and prevention of trichomoniasis and to avoid recurrence [13].

The current diagnostic methods of *T. vaginalis* are mainly microscopic observation [12]. Although these tests have a high specificity, they have a low sensitivity of 40% to 70% [14]. In recent years, the nucleic acid amplification test (NAAT), direct fluorescent antibody test (DFA) and latex agglutination test (LAT) have also been developed for the detection of *T. vaginalis*. However, while these methods greatly improve the sensitivity of the assay, they carry the risk of false positives while making it difficult to meet the requirements of field testing in a non-laboratory environment [15].

CRISPR-Cas (clustered regularly interspaced short palindromic repeats) technology is a new generation of gene editing technology [16]. This technology exhibits nonspecific *trans*-cleavage activity upon binding to its specific target, thus achieving the goal of efficient pathogen-specific detection. To improve the sensitivity of the CRISPR-Cas technique, it is usually combined with nucleic acid amplification methods, such as recombinase polymerase amplification (RPA) [17], loop-mediated isothermal amplification (LAMP) [18] and PCR [19] to preamplify the target genes. The *trans*-cleavage property of the CRISPR-Cas system can compensate for the risk of false-positive nucleic acid amplification methods. Depending on the type of nucleic acid substrate (DNA or RNA), CRISPR-Cas12a/Cas13-based systems have been applied alternatively for the detection of different pathogens, including viruses [20], bacteria [17] and parasites [21].

However, to date, no method has been reported for the detection of *T. vaginalis* using the CRISPR-Cas system. Here, the *actin* gene was chosen as the target site. As the structural protein present in the eukaryotic cytoskeleton, the actin protein is both interspecies specific and intraspecies conserved [22]. Meanwhile, *actin* was also the gene of choice for molecular-typing techniques and diagnosis. In this study, we established a CRISPR-Cas12a-based RPA-LFS assay. The sensitivity of Cas12a detection was enhanced by RPA, and the cleavage effect of CRISPR-Cas12a was used to compensate for the RPA-prone false-positive detection results. In combination with the lateral flow strip (LFS) biosensor, a rapid, sensitive, highly specific, naked-eye visualization method was developed for the rapid detection of *T. vaginalis* in the field.

## Methods

### Nucleic acids, reagents and kits

All primers in this experiment were ordered from Comate Bioscience (Changchun, China). The fluorescent ssDNA reporter (5′6-FAM-TTATT-BHQ1-3′) and lateral flow strip test reporter (5′6-FAM-TTATT-Biotin-3′) were ordered from General Biol Co., Ltd. (Anhui, China). The recombinant plasmid Cas12a (huLbCpf1) was purchased from Addgene (Watertown, MA, USA). The HiScribe™ T7 Quick High Yield RNA Synthesis Kit and NEbuffer 3.1 were purchased from New England Biolabs (MA, USA). The miRNeasy Mini Kit was purchased from QIAGEN (Hilden, Germany). QuickCut *Not*I, QuickCut EcoRI, TaKaRa Taq and RNase inhibitor I were purchased from TaKaRa Bio Inc. (Dalian, China). HindII, MseI and RsaI were purchased from New England Biolabs (Ipswich, MA, USA). The TwistAmp™ basic kit was purchased from TwistDx Ltd. (Hertfordshire, UK). Cas12/13-specific nucleic acid detection kits were purchased from Tiosbio Inc. (Beijing, China). DNA genomes from eight pathogens (*T. vaginalis*, *Candida albicans*, *Mycoplasma hominis*, *Neisseria gonorrhoeae*, *Escherichia coli*, *Cryptosporidium parvum*, *Giardia lamblia* and *Toxoplasma gondii*) and the human genome were provided by our laboratory. The human vaginal secretion, semen and urine genome used for clinical sample testing were sourced from the Second Hospital of Jilin University in Changchun, Jilin Province.

### *Trichomonas vaginalis* culture

Positive clinical samples and *T. vaginalis* standard strain were cultured in glass tubes in liver extraction medium containing 16% fetal bovine serum (BioInd, Israel) and 1% penicillin/streptomycin (BioInd, Israel) [23]. The *T. vaginalis* culture was incubated at 37 °C; every 12 h, it was observed by microscopy for morphology, motility and the presence of worm contamination. The density of *T. vaginalis* was calculated using a cell counting plate, and transmission was performed when the density of *T. vaginalis* reached 10^6^ ml^−1^.

### DNA extraction

The genomic DNA from *T. vaginalis* and clinical samples was extracted by a TIANamp Genomic DNA Kit according to the instructions. DTT (0.1 M) was added when extracting the semen genome and digested at 56 °C for 3 h. Then, the genome was quantified using a Nanodrop and stored at − 20 °C for future experiments.

### Construction of pGEX-4T-1-*actin*-positive recombinant plasmids

PCR primers with homologous recombination sites (underlined bases) were designed based on the *T. vaginalis actin* gene (GenBank accession number: AF237734): TV-*actin*-F: 5′-ccgcgtggatccccggaattcGGCTTCTCTGGCGATGAAGC-3′ and TV-*actin*-R: 5′-ctcgagtcgacccgggaattcCTCCTTGGTGATAACCATCTGTGG-3′.

The 50-μl PCR cocktail contained 2.5 μl of each forward and reverse primer, 25 μl of PrimeSTAR Max Premix, 10 μl of *T. vaginalis* genomic DNA and 10 μl of nuclease-free water. The PCR cycling conditions were as follows: 98 °C for 2 min; 98 °C for 10 s, 55 °C for 5 s, and 72 °C for 1 min for a total of 30 cycles; and a final 72 °C extension for 10 min. Then, the positive PCR product was purified and cloned into the pGEX-4T-1 vector, and a positive recombinant plasmid containing the conserved region was obtained. The DNA concentration was determined using a Nanodrop, and its copy number was calculated according to the formula: copy number (copies μL^−1^) = 6.02 × 10^23^ × Y (ng μL^−1^) × 10^–9^/(number of plasmid bases × 660) (Y denoted plasmid concentration). The DNA was serially diluted tenfold to one copy and stored at − 20 °C for future experiments.

### CRISPR-Cas12a prokaryotic expression and purification

To express the Cas12a protein, the huLbCpf1 gene was cloned from the pET-28a(+) vector into the pGEX-4T-1 vector and transformed into *E. coli* BL21 (DE3). Protein expression was induced at 16 °C for 18–20 h at 150 rpm min^−1^ by 1 mM isopropyl-β-d-thiogalactoside when the optical density at 600 nm reached 0.6. Bacteria were collected and centrifuged at 4 °C at 12,000×*g* for 10 min and then lysed by a sonicator.

The cell lysate was clarified by centrifugation, and the supernatant was collected and transferred to a chromatographic column with a GST agarose gel. The CRISPR Cas12a protein was then eluted with elution buffer (30 ml 1 × PBS, 0.1 g reduced glutathione). The purified protein was identified using SDS-PAGE according to the manufacturer's instructions, decontaminated with a 0.22-μm filter membrane and stored at − 80 °C.

### Design and synthesis of *T. vaginalis* target DNA and crRNA

The *actin* gene was chosen to design target genes and crRNA sequences. Because Cas12a specifically recognizes TTTN sequences, three target sequences (TV1, TV2 and TV3) were designed for the target DNA, as shown in Table 1. Double-stranded DNA (dsDNA) was obtained after annealing, and the dsDNA was quantified by Nanodrop and stored at − 20 °C. The crRNA-F was formed using the T7 promoter (TAATACGACTCACTATAGG) + scaffold sequence of LbCas12a (AATTTCTACTAAGTGTAGAT) + target sequence (20–23 bp after the target PAM sequence), with reverse complementation as crRNA-R, as shown in Table 1. The crRNA was transcribed using a HiScribe™ T7 Quick High Yield RNA Synthesis Kit followed by DNase I digestion and miRNeasy Mini Kit purification. Two microliters was removed to measure the concentration using a Nanodrop, and 2 μl was run on an agarose gel to identify whether crRNA was degraded; the samples were stored in liquid nitrogen.

**Table 1:** Primer sequences for RPA, crRNA and CRISPR-Cas12a

Assay	Primer name	Sequence (5′ → 3′)
Target	TV1	TTTCCCTCTACTCCTCTGGCCGTA
	TV2	TTTCGATGCTGGTGATGGTGTTTC
	TV3	TTTCCCATCCGTTGTTGGCCGTCC
RPA	RPA-F1	CCAAAGGCTAACCGTGAGAAAATGA
	RPA-F2	CATTCAACGCCCCATCCTTCTATGTCGG
	RPA-F3	GGCTGTTCTTTCCCTCTACTCCTCTGGC
	RPA-F4	AACCCAAAGGCTAACCGTGAGAAAATGAT
	RPA-F5	TCCAAGGCTGGTGTCCTCATCCTCAAGTA
	RPA-F6	TAACCCAAAGGCTAACCGTGAGAAAATGA
	RPA-F7	GCTCCAAGGCTGGTGTCCTCATCCTCAAG
	RPA-R1	GAAGTATGGCTTGAAGAGCATTTCTGGGC
	RPA-R2	TAGCCTTCGTAAATTGGAACTGTGTGGGAAAC
	RPA-R3	GGAGTAGCCTTCGTAAATTGGAACTGTGTGGG
	RPA-R4	GGCTGTTGTGTTGAAAGCATTGCCACGCTCTGTG
crRNA	TV1-crRNA	GAAATTAATACGACTCACTATAGGGTAATTTCTACTAAGTGTAGATCCTCTACTCCTCTGGCCGTA
	TV2-crRNA	GAAATTAATACGACTCACTATAGGGTAATTTCTACTAAGTGTAGATGATGCTGGTGATGGTGTTTC
	TV3-crRNA	GAAATTAATACGACTCACTATAGGGTAATTTCTACTAAGTGTAGATCCATCCGTTGTTGGCCGTCC
Cas12a	Cas12a-pGEX-4T-1-F	CCGCGTGGATCCCCGGAATTCATGAGCAAGCTGGAGAAGTTTACA
	Cas12a-pGEX-4T-1-R	TCAGTCAGTCACGATGCGGCCGCGTGCTTCACGCTGGTCTGGG

### RPA primer design and screening

The RPA primers were designed based on the crRNA using Primer Premier 5.0 software (Table 1). According to the TwistAmp™ Basic product specification, the RPA reaction system was 20 μl, including 0.50 μM forward primer, 0.50 μM reverse primer, 1 × rehydration buffer, 14 mM MgOAc, 2 μl genomic DNA of the sample to be tested, and the remaining 20 μl was made up with nuclease-free water. The RPA amplification procedure was performed at a constant temperature of 37 °C for 30 min, and the optimal RPA primers were selected based on the results of nucleic acid electrophoresis.

### RPA-CRISPR-Cas12a detection platform

The RPA-CRISPR-Cas12a platform combined RPA and CRISPR/Cas12a detection. Following the TwistAmp™ Basic product instructions, the RPA reaction mixture was placed at the bottom of the tube. The CRISPR-Cas12a reaction mixture contained 200 nM pGEX-4T-1-Cas12a, 250 nM crRNA, 2 μl DNA target, 20 U RNase inhibitor, 200 nM reporter probe, 20 mM Tris–HCl pH 8.0, 100 mM KCl, 5 mM MgCl_2_, 1 mM DTT, 2.5 μg ml^−1^ glycerol and heparin sodium. Then, 20 μl of the CRISPR-Cas12a reaction mixture was added to the lid of a centrifuge tube and placed in a thermal cycler or water bath at 37 °C for 30 min before sealing. The CRISPR-Cas12a reaction mixture and the RPA reaction mixture were mixed with a short spin. If the ssDNA reporter was 5′6-FAM-TTATT-BHQ1-3′, the centrifuge tube was placed into the EasyPGX quantitative PCR instrument for 1 h at 37 °C, and FAM fluorescence was collected every 2 min. If the ssDNA reporter was 5′6-FAM-TTATT-Biotin-3′, the centrifuge tube was placed into a thermocycler or water bath at 37 °C and incubated for 1 h. Then, the mixtures were drawn and added dropwise to CRISPR-Cas12a/Cas13a-specific test strips to observe the results visually.

### Detection of *T. vaginalis* by *actin* gene-based nested PCR

The *T. vaginalis actin* gene (GenBank: AF237734) was amplified using the outer primers (OPs) and inner primers (IPs) reported in a previous article [24]: OP-F: 5′-TCTGGAATGGCTGAAGAAGACG-3′; OP-R: 5'-CAGGGTACATCGTATTGGTC-3′; IP-F: 5′-CAGACACTCGTTATCG-3′. IP-R: 5′-CGGTGAACGATGGATG-3′.

The reaction mixture (50 μl) included 0.5 µl of TaKaRa Taq, 4 µl of dNTPs, 5 μl of 10 × buffer, 1 µl of each forward and reverse primer, 1.5 µl of genomic DNA template and 37 μl of nuclease-free water. The thermal cycling conditions were: 94 °C for 5 min; 94 °C for 30 s, 58 °C for 30 s and 72 °C for 70 s for a total of 30 cycles; and 72 °C for a 10 min extension. Afterwards, electrophoresis was performed on a 1% agarose gel and visualized under UV light after ethidium bromide staining, and a band at 1100 bp was considered a positive sample.

### *Actin* genotyping of *T. vaginalis* and phylogenetic analysis

The *actin* gene products of positive samples and the *T. vaginalis* standard strain were collected and digested by HindII, MseI and RsaI at 37 °C for 4 h, and eight different *actin* types (A E G H I M N P) were identified according to the location and number of cleavage sites (Table 2) [24]. The digested fragments were separated on a 3% agarose gel by electrophoresis and observed under UV light, and fragment size was assessed. The *actin* genes of samples 3 and 4 and *T. vaginalis* strains were sequenced, and phylogenetic analysis was then performed using the NJ algorithm.

**Table 2:** Size of fragments, pattern groups and *actin* genotypes of the *T. vaginalis* (extracted from [22])

Genotype	HindII (bp)	RsaI (bp)	MseI (bp)
A	827, 213, 60	568, 236, 190, 106	581, 519
E	827, 213, 60	568, 236, 106, 103, 87	581, 315, 204
G	426, 401, 213, 60	568, 236, 190, 106	581, 519
H	426, 401, 213, 60	568, 236, 106, 103, 87	581, 519
I	426, 401, 213, 60	452, 236, 190, 116, 106	581, 519
M	426, 401, 213, 60	568, 236, 190, 106	581, 333, 186
N	426, 401, 213, 60	568, 236, 106, 103, 87	581, 333, 186
P	426, 401, 213, 60	452, 236, 116, 106, 103, 87	581, 333, 186

### RNA extraction

In total, 1 × 10^7^ *T. vaginalis* cultured in vitro for 48 h was collected by centrifugation, and total RNA was extracted using the TRIzol protocol according to the manufacturer’s instructions. Total RNA extracted from trophozoites was analyzed by agarose gel electrophoresis. If a viral band of approximately 5.5 kb was observed, it was regarded as a virus-carrying strain.

### PCR amplification of the ITS fragments

The primers TV-ITS-F and TV-ITS-R [25] (forward primer 5′-ACCGCCCGTCGCTCCTACCGA-3′ and reverse primer 5′-CTCCGCTTAATGAGATGCTTC-3′) were used to amplify the ITS region of ribosomal DNA (rDNA) from clinical sample 4. The reactions were carried out in a total volume of 50 µl and included 0.5 µl of TaKaRa Taq, 4 µl of dNTPs, 5 μl of 10 × buffer, 1 µl of each forward and reverse primer, 1 µl of genomic DNA template and 37.5 μl of nuclease-free water. The thermal cycling conditions were: 94 °C for 5 min; 94 °C for 30 s, 58 °C for 30 s and 72 °C for 70 s for a total of 30 cycles; and 72 °C for a 10 min extension. PCR products were purified using a PCR Product Purification Kit and sent to Comate Bioscience (Changchun, China) for sequencing.

### Statistical analysis

Statistical analyses were performed using GraphPad Prism, and one-factor analysis of variance (ANOVA) was used to compare the two groups and the different groups. All experiments were repeated at least three times, and data are shown as mean ± standard error. Differences were considered statistically significant when the *p*-value was < 0.05.

## Results

### Preparation and feasibility of the proposed detection strategy

**Fig. 1:**
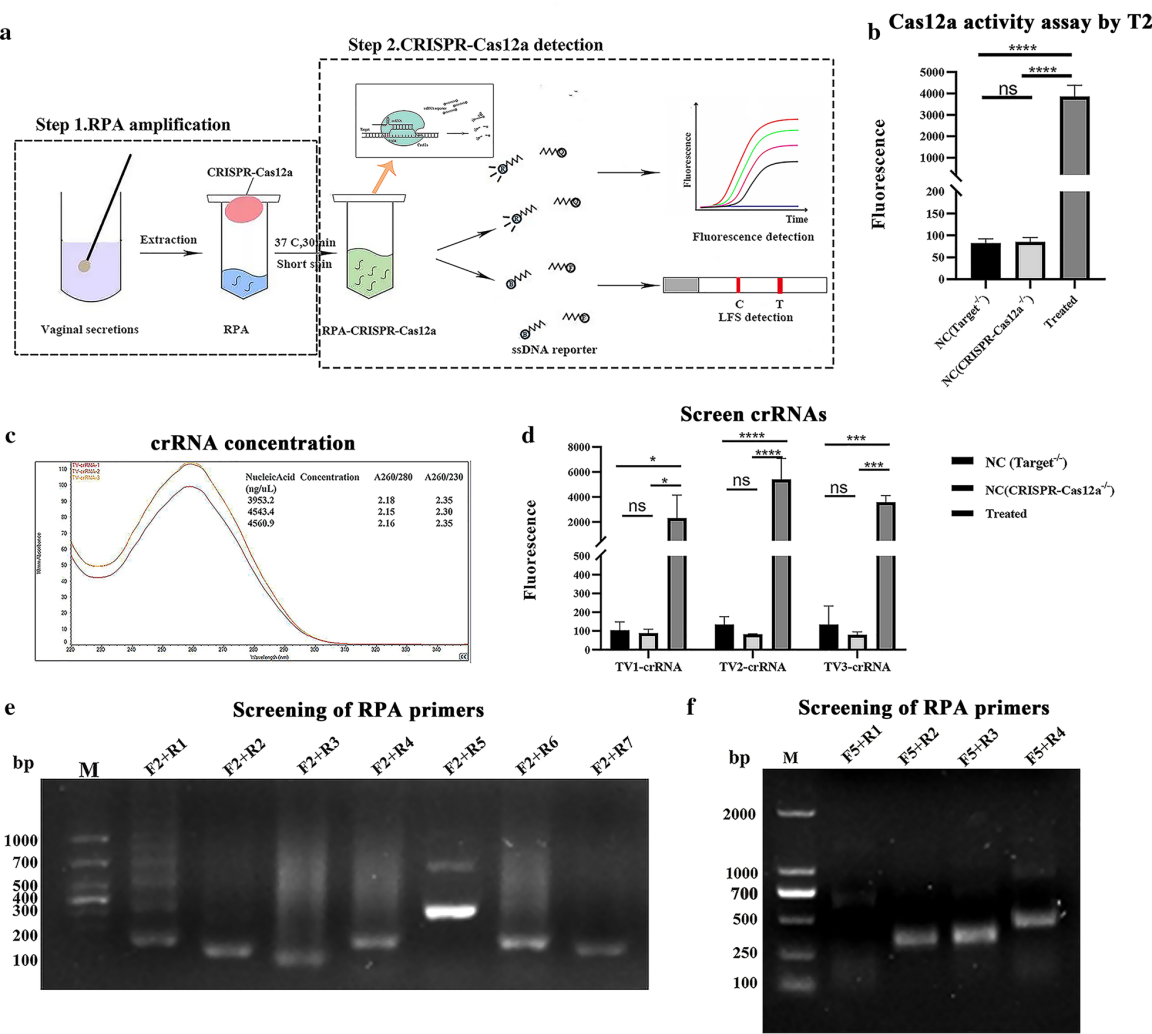
An RPA-CRISPR-Cas12a platform for the detection of *T. vaginalis*. **a** A flow diagram of the RPA-CRISPR-Cas12a detection system in vaginal secretions. DNA extracted from vaginal secretions was preamplified by RPA and mixed with the CRISPR-Cas12a detection system to interpret the results by the fluorescence or lateral flow strip method. **b** CRISPR-Cas12a activity was determined using the T2 sequence as a target (*n* = 3 technical replicates; ****p < 0.0001; bars represent the means ± SEM). **c** Absorbance curves of three purified crRNAs. **d** Screening of crRNAs for *T. vaginalis* DNA detection by Cas12a collateral detection (*n* = 3 technical replicates; *P < 0.05; ***P < 0.001; ****P < 0.0001; bars represent the means ± SEM).** e** Screening for the best RPA forward primers. **f** Screening of RPA reverse primers using preferred forward primers

The biosensor designed in this study was based on the *trans*-cleavage of the CRISPR-Cas12a system and the rapid amplification of RPA. The detection process is shown in Fig. 1a. DNA samples extracted from vaginal swabs were added to the RPA mixture at the bottom of the centrifuge tube, whereas the CRISPR mixture was located on the cap of the tube. After 30 min, the CRISPR mixture was mixed with the amplification product with a short spin. The amplicon could be recognized by specific crRNA fragments, while the reaction activated the *cis*- and *trans*-nuclease activity of Cas12a. When the 5′6-FAM-TTATT-BHQ1-3′ reporter was used, the reporter molecule modified by fluorescent and burst groups was cleaved and released a fluorescent signal. On the other hand, if the 5′6-FAM-TTATT-biotin-3′ reporter was used, the FAM-modified groups specifically bound gold-NP anti-FAM antibodies to form a complex that was captured by the antibody detection line. Biotin-modified groups could be captured by the streptavidin line (control band). When the ssDNA reporter was not degraded by LbCas12a, this complex could be captured by the streptavidin line. In contrast, when ssDNA was degraded, biotin was released from the complex captured by the streptavidin line (control band), and the remaining complex was bound by the antibody capture line (sample band). There was no need to open the lid during the entire assay, and only a reaction condition of 60 min at 37 °C was needed.

### RPA-CRISPR-Cas12a platform

Cas12a protein was produced by the *E. coli* expression system and expressed mainly in soluble form with a molecular weight of ~ 150 kDa, as confirmed by sodium dodecyl sulfate‒polyacrylamide gel electrophoresis (Additional file 1: Fig. S1). To demonstrate that the recombinant Cas12a protein had cleavage activity, the target site T2 was selected [26] to evaluate Cas12a activity. The results showed that the T2 group had a clear fluorescent signal compared with the Target^−/−^ or Cas12a^−/−^ negative control groups [ANOVA, *F*_(2,6)_ = 625.6, *P* < 0.0001] (Fig. 1b).

To obtain efficient and specific crRNAs applicable for the detection of different *T. vaginalis* isolates, three groups of targets were selected, and their corresponding crRNA oligonucleotide single strands are shown in Table 1. Three sets of crRNAs were of high concentration and purity (Fig. 1c) and were not degraded after transcription (Additional file 1: Fig. S2). Next, the efficiencies of the three candidate crRNAs were tested using a fluorescence detector. The results showed that TV1-crRNA, TV2-crRNA and TV3-crRNA all had clear fluorescent signals compared with the negative controls (Target^−/−^ or Cas12a^−/−^) (Additional file 1: Fig. S3). However, TV2-crRNA showed a higher fluorescence signal than the other crRNAs [TV1-crRNA: ANOVA, *F*_(2,6)_ = 9.615, *P* = 0.0135, TV2-crRNA: ANOVA, *F*_(2,6)_ = 1968, *P* < 0.0001, TV3-crRNA: ANOVA, *F*_(2,6)_ = 36.94, *P* = 0.0004] (Fig. 1d).

Finally, to improve the sensitivity of the RPA-CRISPR-Cas12a platform, we screened the best RPA primer results and showed that the target gene products of these primers could be observed, especially RPA-F5 and RPA-R3, which showed strong target bands and the highest amplification efficiency (Fig. 1e, f).

### Specificity and sensitivity testing of RPA-CRISPR-Cas12a

**Fig. 2:**
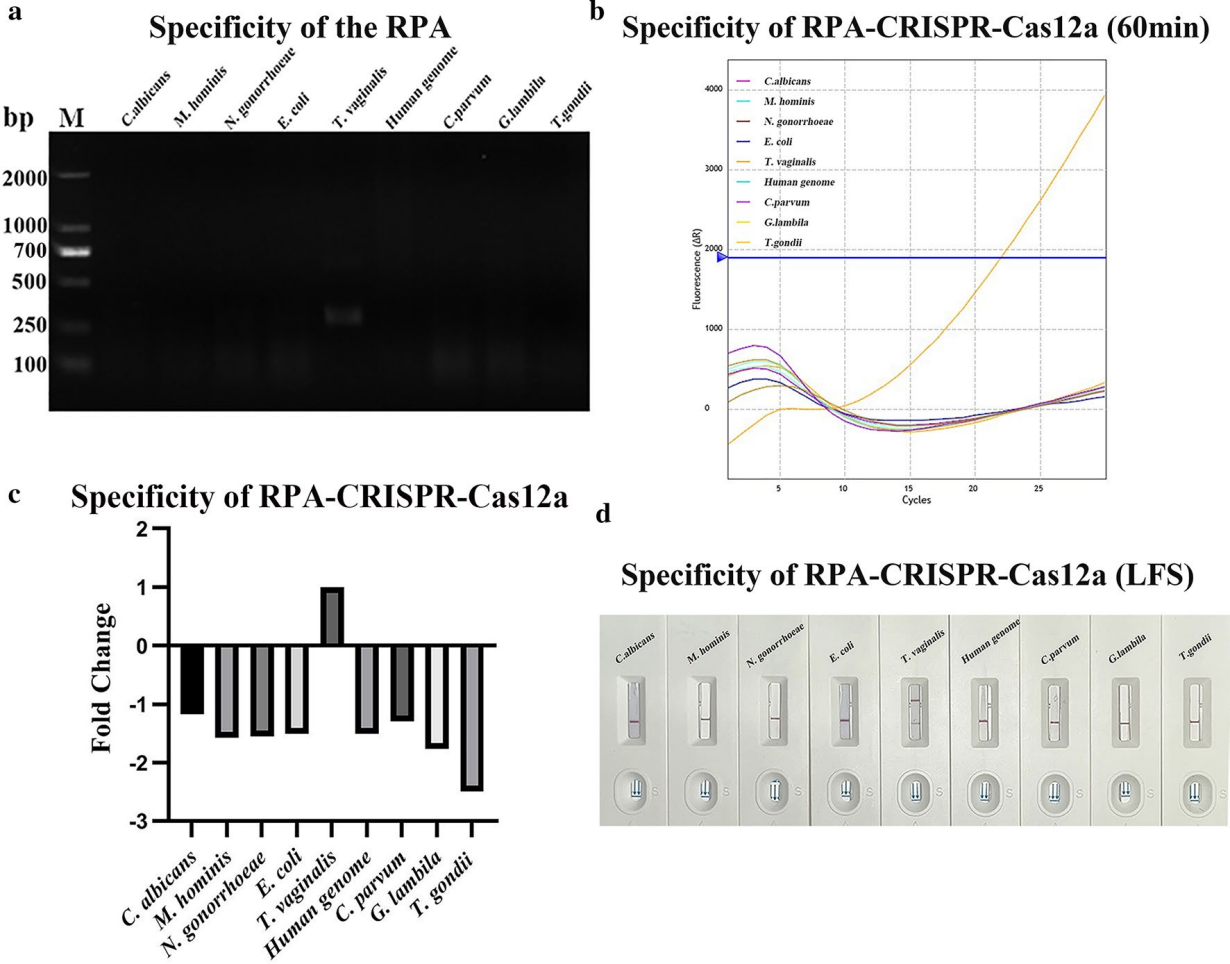
Specificity of the RPA-CRISPR-Cas12a detection platform. **a** Agarose gel electrophoresis of the genomes of *Trichomonas vaginalis*, eight pathogens (*C. albicans, M. mycoplasma, N. gonorrhoeae, E. coli, C. parvum*, *G*. *lambila* and *T. gondii*) and the human genome after RPA amplification. **b** Specificity of the RPA-CRISPR-Cas12a platform for the detection of *T. vaginalis* by fluorescence. **c** The fluorescence multiplicity change (FC) of eight pathogens, the human genome and *T. vaginalis*, FC = (F (PC) − B (PC))/(F (NC) − B (NC)). **d** Specificity of the RPA-CRISPR-Cas12a platform for the detection of *T. vaginalis* by the LFS sensor

To demonstrate the sensitivity and specificity of the RPA-CRISPR-Cas12a platform, genomes from *C. albicans*, *M. hominis*, *N. gonorrhoeae*, *E. coli*, the human genome, *C. parvum*, *G. lamblia* and *T. gondii* were used for comparative tests. The results showed that only the *T. vaginalis* genome produced positive results after amplification by RPA (Fig. 2a). The same results were observed in the RPA-CRISPR-Cas12a platform (Fig. 2b, d). In the fluorescence reporting test, we recorded fluorescence signal values once every 2 min for 60 min. We defined the 0 min fluorescence signal value as B and the 60 min fluorescence signal value as F. In each test, the value of the fold change (FC) was generated as FC = (F (PC) −  B (PC))/(F (NC) − B (NC)). The results showed that only the *T. vaginalis* genome had a positive fluorescence fold change, while the rest of the genomes were negative (Fig. 2c).

**Fig. 3:**
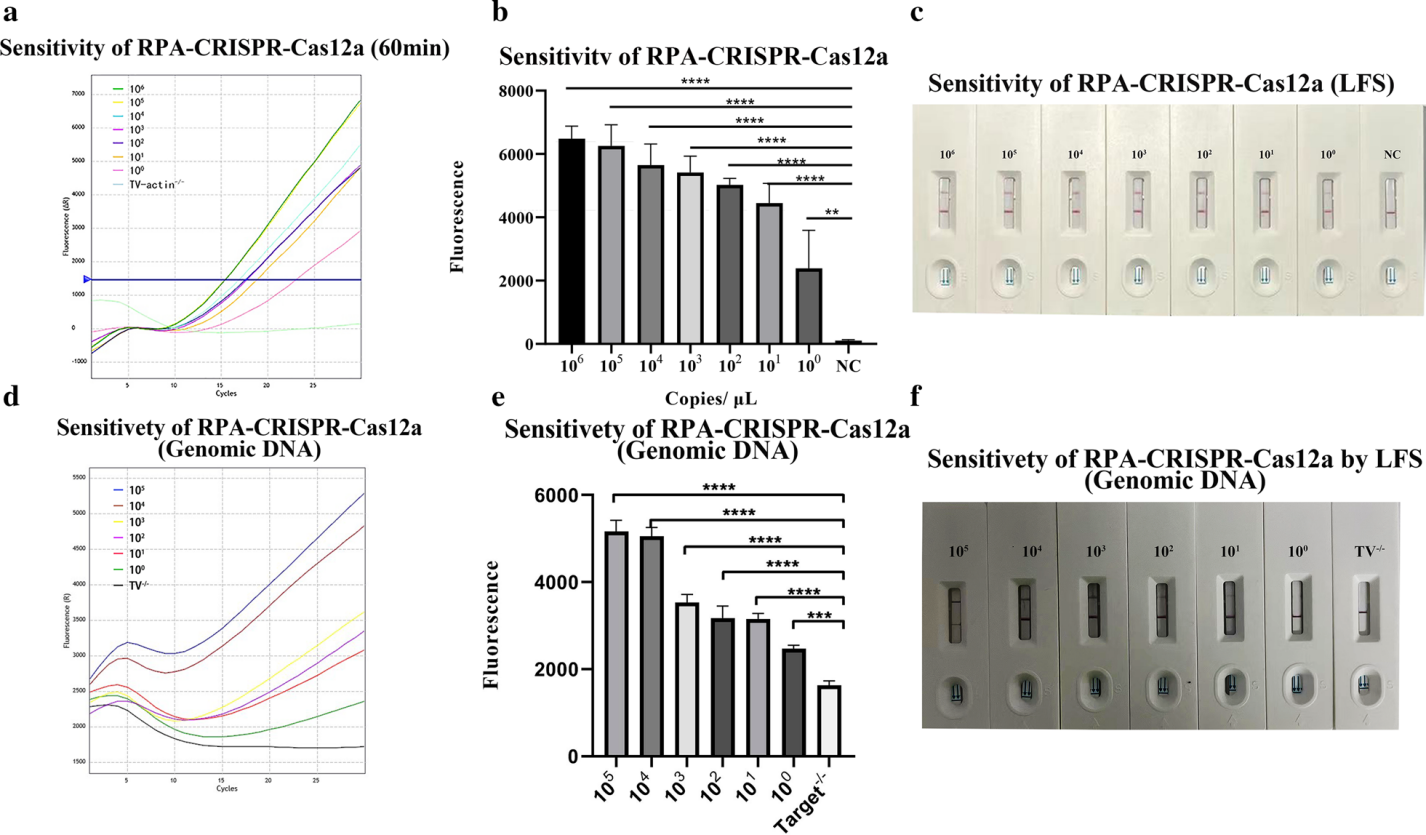
Sensitivity of the RPA-CRISPR-Cas12a detection platform. **a** Sensitivity of the RPA-CRISPR-Cas12a platform to fluorometric assays using recombinant plasmids. TV-*actin*^−/−^ was used as a negative control. **b** RPA-CRISPR detected 10^0^ copies of the *actin* gene by the Cas12a excision reaction within 60 min (*n* = 3; ****P < 0.0001; **P < 0.01; bars represent the means ± standard error of the mean). **c** Sensitivity of the RPA-CRISPR-Cas12a platform to the LFS sensor using recombinant plasmids. NC was used as a negative control. **d** Sensitivity test of the fluorescence assay using crude DNA extracted from *T. vaginalis*. **e** RPA-CRISPR detected one *T. vaginalis* by Cas12a excision reaction within 60 min (*n* = 3; ****P < 0.0001; ***P < 0.01 bars represent the means ± standard error of the mean). **f** Sensitivity test of crude DNA extracted from *T. vaginalis* by the LFS sensor

To evaluate the sensitivity of the RPA-CRISPR-Cas12a detection platform, a pGEX-4T-1-*actin* plasmid was constructed and applied to this platform. The results of RPA amplification analysis by agarose gel electrophoresis showed that ten copies and above were able to detect positive results, while the one-copy DNA bands were fainter (Additional file 1: Fig. S4). However, with the RPA-CRISPR-Cas12a platform, positive results were observed at one copy number and above with both the fluorescent reporter (Fig. 3a) and the LFS biosensor (Fig. 3c). With serial dilutions, a limit of one copy of pGEX-4T-1-*actin* could be detected with TV2-crRNA [10 copies and above: ANOVA, *F*_(7,16)_ = 35.81, *P* < 0.0001, 1 copy: ANOVA, *F*_*(*7,16)_ = 35.81, *P* = 0.0024] (Fig. 3b).

The sensitivity of the RPA-CRISPR-Cas12a assay platform was also tested with genomic DNA from *T. vaginalis*. Prior to DNA extraction, the concentration of *T. vaginalis* was adjusted to 1 × 10^5^ parasites per milliliter. Extracted DNA was serially diluted to one parasite per milliliter. In the fluorescence reporter assay, samples with a parasite count of one or higher showed an observable fluorescence signal (Fig. 3d and e). In the LFS biosensor, samples with ten parasites showed weaker test lines, indicating that ten parasites per milliliter can be detected based on LFS (Fig. 3f).

### Detection of clinical samples by an RPA-CRISPR-Cas12a-based assay and nested PCR

**Fig. 4:**
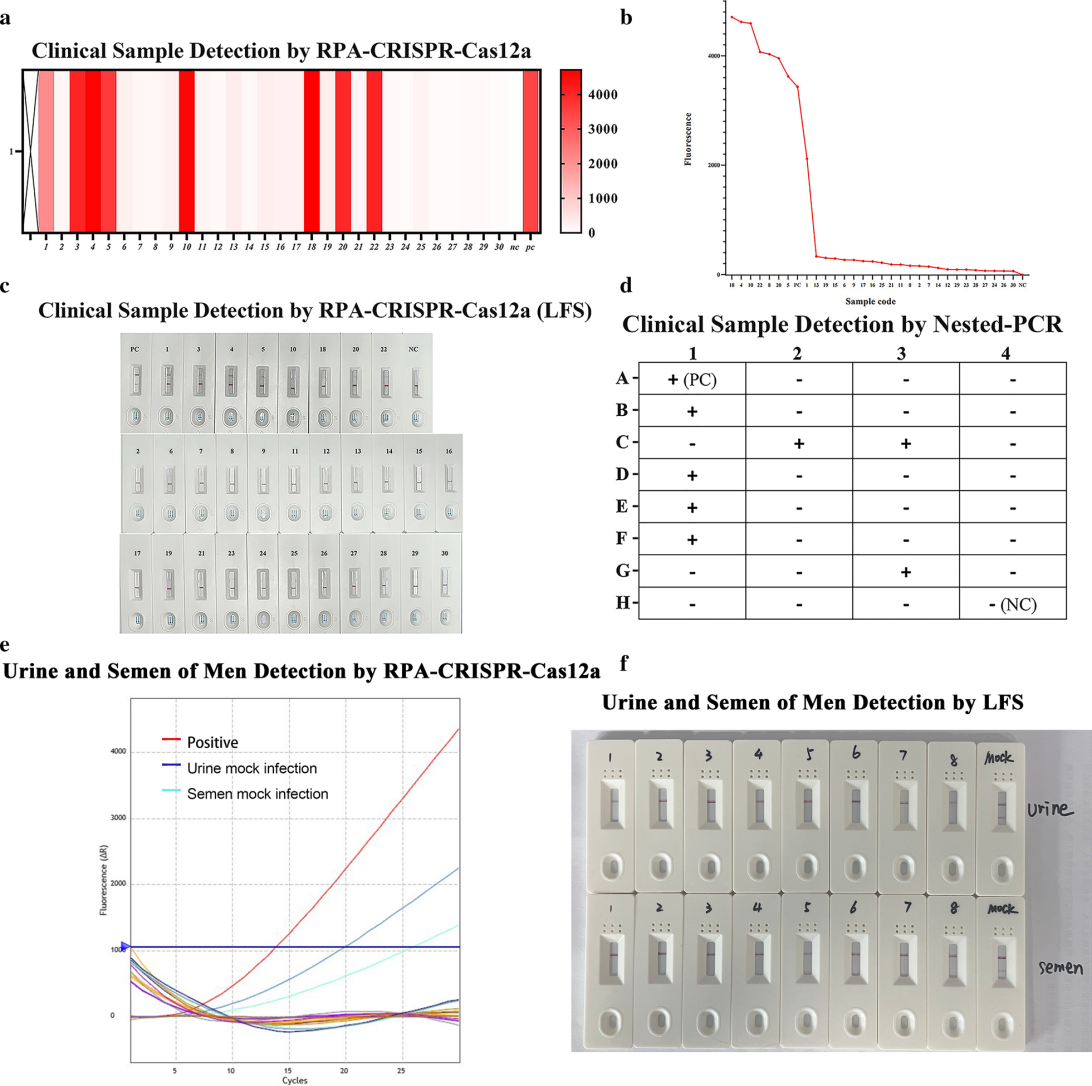
**a** Thermogram of 30 clinical samples (vaginal swabs). Sample colors represent fluorescent signals; dark colors are positive, and light colors are negative. **b** Thirty clinical samples (vaginal swabs) with a relative fluorescence intensity > 400 were considered positive. **c** RPA-CRISPR-Cas12a assay platform results of 30 clinical samples (vaginal swabs) were interpreted using an LFS sensor. **d** Detection of clinical samples by conventional nested PCR. **e** Detection of 18 male samples by fluorescent sensors. **f** RPA-CRISPR-Cas12a assay platform results of 19 male samples were interpreted using an LFS sensor. MOCK indicates a laboratory mock infection sample

To evaluate the clinical detection effectiveness of the RPA-CRISPR-Cas12a assay platform, DNA from 30 human vaginal swabs with good purity (OD260/OD280 = 1.8–2.0) was collected (Additional file 1: Fig. S5) and tested using the RPA-CRISPR-Cas12a assay platform. Based on the sensitivity results, 60 min was chosen as the reaction time of the CRISPR-Cas system to avoid false negatives. The results were interpreted using the fluorescent reporter (Fig. 4a, b and Additional file 1: Fig. S6) and the LFS biosensors (Fig. 4c), respectively; 26.7% (8/30) of the samples were *T. vaginalis*-positive by these two assays.

Meanwhile, these 30 samples were tested using the established nested PCR assay (Additional file 1: Fig. S7), and the positive rate of *T. vaginalis* was 23.3% (7/30) (Fig. 4d). Interestingly, sample 20 was found to be *T. vaginalis* positive using the RPA-CRISPR-Cas12a assay platform but failed to be detected using the nested PCR assay. The rest of the positive samples in the nested PCR assay were consistent with those in the RPA-CRISPR-Cas12a assay. Subsequently, we sequenced the RPA amplification product of sample 20 and found the sequence to be a partial sequence of the *T. vaginalis actin* gene.

We extracted eight male urine genomes and eight semen genomes and set up one urine mock infection sample and one semen mock infection sample as controls. The RPA-CRISPR-Cas12 assay platform was used for testing, and the fluorescence and LFS results showed that the mock-infected samples were positive, and the clinical samples were all negative (Fig. 4e, f). These 18 samples were also tested using nested PCR, and the results were consistent with the results of the RPA-CRISPR-Cas12 assay platform (Additional file 1: Fig. S8).

### *Actin *gene-based molecular typing of *T. vaginalis* clinical isolates

**Fig. 5:**
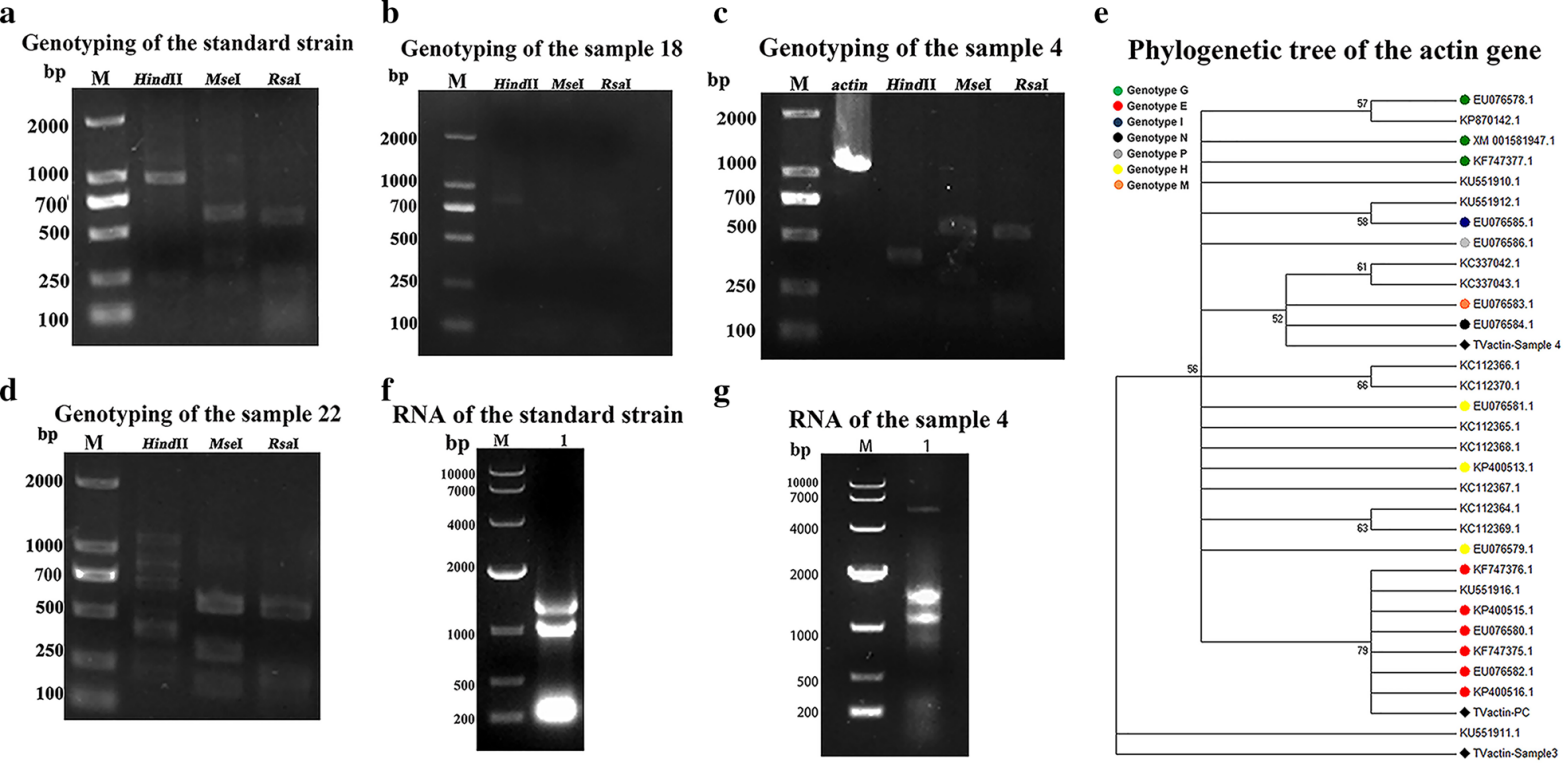
Genotyping and virus determination of the *T. vaginalis* standard strain and clinical strains. **a** Agarose gel electrophoresis for genotyping of the *T. vaginalis* standard strain. **b** Agarose gel electrophoresis for genotyping of the sample 18. **c** Agarose gel electrophoresis for genotyping of sample 4; *actin* was a nonenzymatic control. **d** Agarose gel electrophoresis for genotyping sample 22. **e** Phylogenetic tree of the *actin* gene of *T. vaginalis*. The sequences detected in this study are indicated by the diamond shape. The other sequences shown in the tree were reference genotypes collected from GenBank. The scale bar shows the nucleotides per 1000 substitutions. **f** Agarose gel electrophoresis of total RNA of the *T. vaginalis* standard strain (TVV^−/−^). **g** Agarose gel electrophoresis of total RNA of clinical isolate 4 (TVV)

To analyze the type of *T. vaginalis* isolated from clinical samples, the *actin* genes of samples 1, 3, 4, 5, 18 and 22 and the *T. vaginalis* standard strain were selected for digestion and typing (Table 2). Digestion of nested PCR products of the *T. vaginalis* standard strain showed three digested fragments of 827 bp, 213 bp and 60 bp with HindII; three digested fragments of 581 bp, 315 bp and 204 bp with MseII; three digested fragments of 568 bp, 236 bp and 87 bp with RsaII, indicating that the *T. vaginalis* standard strain was genotype E (Fig. 5a). Sample 18 showed similar digestion results and was also regarded as genotype E (Fig. 5b). Digestion of nested PCR products of sample 4 showed three digested fragments of 426 bp, 213 bp and 60 bp with HindII, two digested fragments of 581 bp and 186 bp with MseII and three digested fragments of 568 bp, 236 bp and 190 bp with RsaII, indicating that sample 4 was genotype M (Fig. 5c). Digestion of nested PCR products of sample 22 showed five digested fragments of 827 bp, 426 bp, 401 bp, 213 bp and 60 bp with HindII; three digested fragments of 581 bp, 315 bp and 204 bp with MseII and three digested fragments of 568 bp, 236 bp and 106 bp with RsaII, indicating that sample 22 was a mix of genotypes N and E (Fig. 5d). Digestions of samples 1, 3 and 5 had the same digested fragments identified, but no reported genotypes corresponded to them (Additional file 1: Fig. S9). In addition, genotypes A, G, H, I and P were not found in these samples.

The *actin* gene of samples 3 and 4 and the *T. vaginalis* standard strain were selected for further sequence analysis. Compared with the *T. vaginalis* standard strain, the sequence homology of sample 3 was 99.1%, and the sequence homology of sample 4 was 98.7%. To assess the genetic diversity among *T. vaginalis* isolates, we performed multiple comparisons of various *T. vaginalis actin* genotypes with the sequences found in this study and in GenBank. Phylogenetic analysis showed that the *T. vaginalis* standard strain (TV *actin*-PC) was closely related to KP400516.1, which was regarded as genotype E. Sample 4 was closely related to EU076583.1, which was regarded as genotype M, and sample 3 was closely related to KU551911.1 (Fig. 5e).

RNA of sample 4 and the *T. vaginalis* standard strain was identified by agarose gel electrophoresis, and the results showed that, compared with the *T. vaginalis* standard strain (TVV^−/−^), there was an obvious band at 5000 bp, which was initially judged to be a *T. vaginalis* strain with virus in sample 4 (Fig. 5f, g). Meanwhile, we amplified the internal transcribed spacer (ITS) of clinical isolate 4 for sequence analysis, and the results showed the highest homology rate of 99.8% with TVI:MCP (ATCC 30236), which was a virus-containing strain. These data indicated that sample 4 was a *T. vaginalis* strain with virus.

## Discussion

The incidence of trichomoniasis among women in the US exceeds that of *Neisseria gonorrhoeae* and *Chlamydia trachomatis* infections combined [27], and the annual medical costs associated with trichomoniasis have approached $34 million. Moreover, in a cross-sectional study conducted in Yunnan and Henan Provinces, China, the prevalence of *T. vaginalis* was 9.0% and 1.6%, respectively, and clinically positive samples were observed by microscopy [22]. Efficient and accurate diagnostic tools are particularly important for mass population screening and control of *T. vaginalis* as well as for surveillance in public health facilities. The current main clinical diagnostic method for *T. vaginalis* is microscopic observation, which is simple and time-saving. However, it is not suitable for inexperienced inspectors because the sensitivity of the test is unstable, and the detection rate is subject to fluctuations. Therefore, we must develop a diagnostic procedure that satisfies all of the following criteria: sensitive, fast, robust, requires no equipment and can be deliverable to the user.

As a specific nucleic acid recognition tool, detection methods based on the CRISPR-Cas system have been widely developed in recent years. In this study, an RPA-CRISPR-Cas12a for the rapid detection of *T. vaginalis* was established and can be used in clinical practice. First, the method can be applied to analyze fluorescence detection samples and can further be combined with LFS to visualize the results with the naked eye, which does not require specialized technicians or expensive equipment. Thus, it is suitable for large-scale field detection and can even be implemented at home by itself. Second, the combination of RPA amplification and CRISPR makes the highly sensitive Cas12a detection more specific, which avoids the loss of trace nucleic acids [19], compensates for the disadvantages of RPA false-positives, amplifies the cleavage signal of CRISPR and reduces dye contamination generated during staining when compared to RPA or PCRs alone. Third, the RPA-CRISPR-Cas12a diagnostic platform developed in this study is a closed one-pot reaction. It was compared to the use RPA/RAA as the first preamplification step for detecting pathogens, such as ZIKV [20], swine fever virus [28], *T. gondii* [21] and *C. parvum* [15], which are closed one-pot reactions without opening the lid throughout the experiment. This procedure can minimize contamination and false-positive findings and save time. The PRA-CRISPR-Cas12a assay platform established in this study is time-saving and economical. Clinical sample preparation took 30 min or 180 min (including sperm digestion time) and cost approximately $1.00. The amplification reaction took 20 min and cost $2.60. The assay procedure cost $1.4 using the LFS assay and only $0.2 using the fluorescence assay, and the results could be observed in as little as 20–30 min.

Although the trans-cleavage activity of CRISPR does not create a detectable signal at low target gene concentrations (< 10 nM), single-molecule detection under preamplified conditions can be obtained [17]. Comparative analysis of several amplification methods showed that the PCR procedure is complex, requires a thermocycler and has a long amplification time. The primer design of the LAMP approach is relatively complicated, and the amplification temperature of LAMP is 60–65 °C, which is far from the ideal temperature of the CRISPR-Cas detection system. Nevertheless, the RPA reaction has the advantages of being quick, having a high amplification sensitivity and being able to perform multiplex reactions [29].

It has been observed that *T. vaginalis* carrying virus (TVV) increases *T. vaginalis* toxicity, upregulates proinflammatory responses in the host and increases *T. vaginalis* adherence to host cells [1]. Simultaneously, it has been shown that the etiology of *T. vaginalis* resistance to metronidazole, the preferred treatment for trichomoniasis, is associated with multiple single nucleotide alterations in the *actin* gene [24, 30]. Since the genotype of *T. vaginalis* and whether this parasite carries TVVs play important roles in the pathogenesis and drug resistance of *T. vaginalis* [24], identifying the genotype of the parasite and the existence of TVVs can help provide a theoretical foundation for parasite control. We used the target gene to compare with the t seven genotypes of *T. vaginalis* (E, G, H, I, M, N and P) (Additional file 1: Fig. S10). The results showed that the target gene selected in this test were highly conserved among the seven genotypes. However, the prevalence of genotype A of *T. vaginalis* is very low, and no known sequence has been found for alignment. Therefore, we believe that the method of this study was applicable to most genotypes. We discovered that the clinical samples of *T. vaginalis* were genotyped E, M or N. In some previous studies, genotypes E and G were reported as the most prevalent, but they were detected in both TVV and TVV^−/−^ samples, while the distribution of virus-carrying strains in the phylogenetic tree was random and not in the same cluster. This suggests that TVV may infect virus-free strains and that there is no relationship between the presence or absence of virus in the parasite and the genealogical status of the parasite [31]. In addition, we found a strain of *T. vaginalis* carrying the virus, which implied that the treatment and control of this *T. vaginalis* strain may be more difficult than those of the *T. vaginalis*-only strain.

In the future, we can establish a multiplexed platform using the CRISPR-Cas system that relies on the specific cleavage preferences of the Cas enzyme, depending on the needs for *T. vaginalis* detection, prevention and control, where several target molecules need to be identified in a single reaction. For example, FAM, HEX, Texas Red, Cy5 and Quasar 705 channels can be used in the same reaction and simultaneously combined with LwaCas13a, PsmCas13b, CcaCas13b and Cas12a [17] to detect *T. vaginalis* and TVV, as well as genotype alleles in *T. vaginalis*, resulting in a high-throughput multiplexed assay platform. The next step is to make the signal output from the RPA-CRISPR-Cas12a diagnostic platform more quantitative, sensitive and stable by optimizing the buffer composition and concentration and crRNA design to optimize the reaction and by lyophilizing the CRISPR system components for storage. We could even combine a portable glucose meter (PGM) or microfluidic chip to establish a PGM or microfluidic chip-based digital assay for *T. vaginalis* gene nucleic acid quantitation.

## Conclusions

In conclusion, the RPA-CRISPR-Cas12a single-tube *T. vaginalis* field test platform developed in this study can be used as a clinical detection tool for *T. vaginalis* with low equipment requirements, is especially suitable for large-scale population screening and can provide a good technical tool for surveillance in public health institutions.

## Supplementary Information

**Additional file 1: Fig. S1.** Expression, purification, and identification of pGEX-4sT-1-Cas12a. **Fig. S2.** Agarose gel electrophoresis of three purified crRNA nucleic acids. **Fig. S3.** Screening of crRNA for *T. vaginalis* detection by CRISPR-Cas12a. Cas12a^−/−^ and T2^−/−^ were negative control. **Fig. S4.** Agarose gel electrophoresis of the pGEX-4T-1-*actin* positive plasmid after amplification with RPA. **Fig. S5.** Absorbance values of 30 clinical samples by Nanodrop. **Fig. S6.** Clinical samples detection by RPA-CRISPR-Cas12a. **Fig. S7.** Agarose gel electrophoresis of 30 human vaginal secretions by nested PCR. **Fig. S8.** (a) Agarose gel electrophoresis of 9 male urine and semen samples by nested PCR. **Fig. S9.** Molecular typing of clinical positive samples based on the actin gene. **Fig. S10.** Sequence alignment of target gene with different genotypes of *T. vaginalis actin* gene.

## Data Availability

The data that support the findings of this study are available from the correspondence author, Pengtao Gong: gongpt@jlu.edu.cn.

## Abbreviations

HIV: Human immunodeficiency virus
RPA: Recombinase polymerase amplification
LFS: Lateral flow strip
STI: Sexually transmitted infections
NAAT: Nucleic acid amplification test
DFA: Direct fluorescent antibody test
LAT: Latex agglutination test
CRISPR-Cas: Clustered regularly interspaced short palindromic repeats
LAMP: Loop-mediated isothermal amplification
ITS: Internal transcribed spacer
TVV: *Trichomonas vaginalis* virus
PGM: Portable glucose meter
SDS-PAGE: Sodium dodecyl sulfate-polyacrylamide gel electrophoresis
