# Correction: Establishment and application of a CRISPR-Cas12a-based RPA-LFS and fluorescence for the detection of *Trichomonas vaginalis*

**DOI:** 10.1186/s13071-022-05551-w

**Published:** 2022-11-15

**Authors:** Shan Li, Xiaocen Wang, Yanhui Yu, Songgao Cao, Juan Liu, Panpan Zhao, Jianhua Li, Xichen Zhang, Xin Li, Nan Zhang, Min Sun, Lili Cao, Pengtao Gong

**Affiliations:** 1 State Key Laboratory for Zoonotic Diseases, College of Veterinary Medicine, Jilin University, Changchun, 130062 China; 2 Clinical Laboratory, The Second Hospital of Jilin University, Changchun, 130000 China; 3 Pingdu People’s Hospital, Qingdao, 266700 China; 4 Jilin Academy of Animal Husbandry and Veterinary Medicine, Changchun, 130062 China

## Correction: Parasites & Vectors (2022) 15:350 https://doi.org/10.1186/s13071-022-05475-5

Following publication of the original article [1], the authors have updated the Results of the article with the following Genbank numbers: OP557698 and 4 OP557697. Specifically, this information has been added to the second paragraph of subsection ‘Actin gene‑based molecular typing of *T. vaginalis* clinical isolates’, with the sentence ‘The actin gene of samples 3 (OP557698) and 4 (OP557697) and the *T. vaginalis* standard strain were selected for further sequence analysis.’

The authors thank you for reading this update.
