# Supplementary material for: Establishment and application of a CRISPR-Cas12a-based RPA-LFS and fluorescence for the detection of Trichomonas vaginalis
Source: Parasit Vectors. 2022 Sep 30;15:350. doi: 10.1186/s13071-022-05475-5 (PMC9526244; doi:10.1186/s13071-022-05475-5)
Supplement: Supplementary file 1 — Additional file 1: Fig. S1. Expression, purification, and identification of pGEX-4sT-1-Cas12a. Fig. S2. Agarose gel electrophoresis of three purified crRNA nucleic acids. Fig. S3. Screening of crRNA for T. vaginalis detection by CRISPR-Cas12a. Cas12a−/− and T2−/− were negative control. Fig. S4. Agarose gel electrophoresis of the pGEX-4T-1-actin positive plasmid after amplification with RPA. Fig. S5. Absorbance values of 30 clinical samples by Nanodrop. Fig. S6. Clinical samples detection by RPA-CRISPR-Cas12a. Fig. S7. Agarose gel electrophoresis of 30 human vaginal secretions by nested PCR. Fig. S8. (a) Agarose gel electrophoresis of 9 male urine and semen samples by nested PCR. Fig. S9. Molecular typing of clinical positive samples based on the actin gene. Fig. S10. Sequence alignment of target gene with different genotypes of T. vaginalis actin gene. [file 13071_2022_5475_MOESM1_ESM.docx]

**Supplementary Materials for**

**Establishment and application of a CRISPR-Cas12a-based RPA-LFS for Detection of *Trichomonas vaginalis***

Additional file 1: **Fig. S1.** Expression, purification, and identification of pGEX-4T-1-Cas12a. **Fig. S2.** Agarose gel electrophoresis of three purified crRNA nucleic acids. **Fig. S3.** Screening of crRNA for *T. vaginalis* detection by CRISPR-Cas12a. Cas12a^-/-^ and T2^-/-^ were negative control. **Fig. S4.** Agarose gel electrophoresis of the pGEX-4T-1-*actin* positive plasmid after amplification with RPA. **Fig. S5.** Absorbance values of 30 clinical samples by Nanodrop. **Fig. S6.** Clinical samples detection by RPA-CRISPR-Cas12a. **Fig. S7.** Agarose gel electrophoresis of 30 human vaginal secretions by nested PCR. **Fig. S8.** (a) Agarose gel electrophoresis of 9 male urine and semen samples by nested PCR. **Fig. S9.** Molecular typing of clinical positive samples based on the actin gene. **Fig. S10.** Sequence alignment of target gene with different genotypes of *T. vaginalis* *actin* gene.


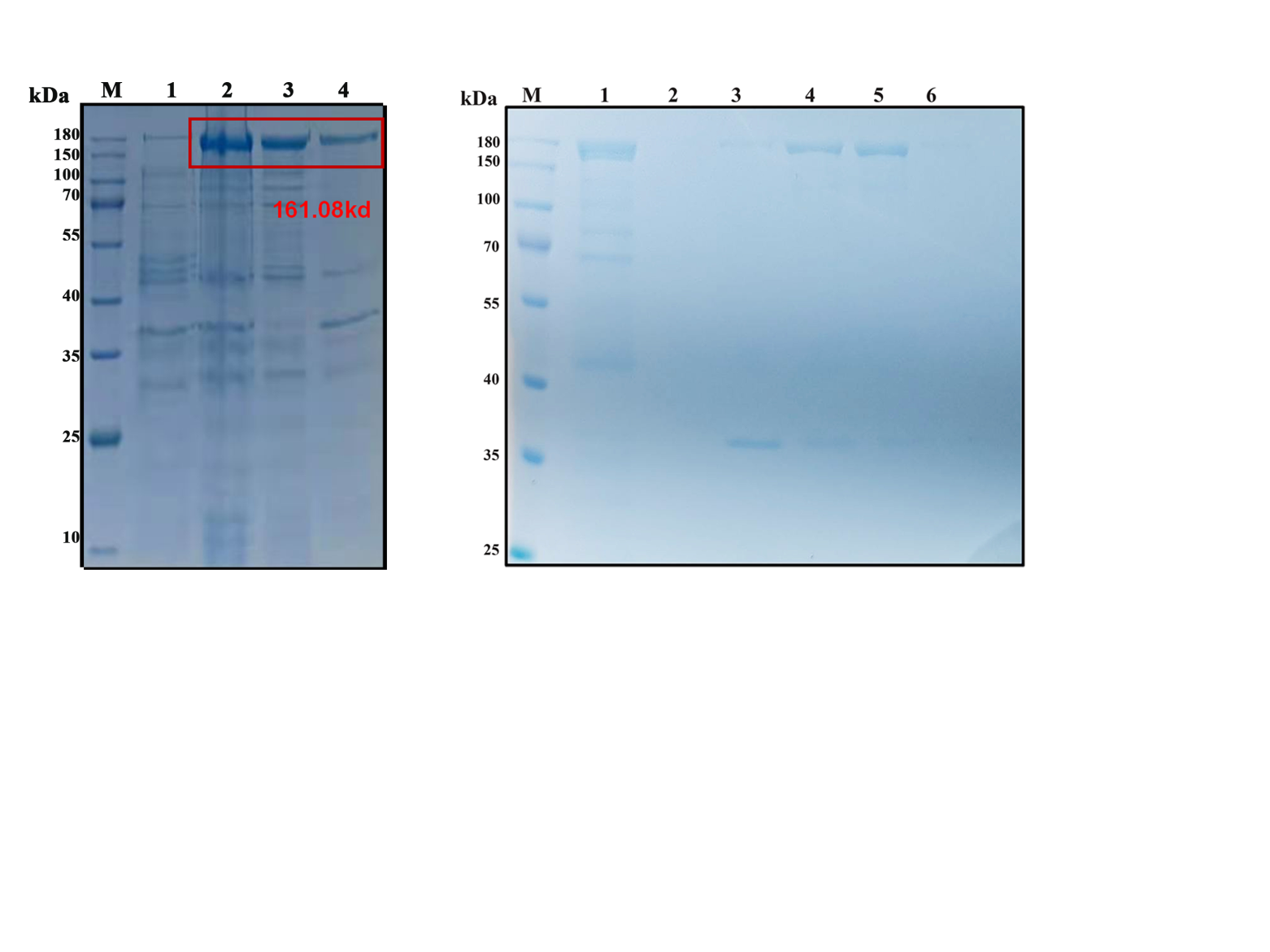


**Fig. S1.** Expression, purification, and identification of pGEX-4T-1-Cas12a. **(a)** Sodium dodecyl sulfate–polyacrylamide gel electrophoresis analysis of pGEX-4T-1-Cas12a expression. lane 1 was lysate of non-induced recombinant bacteria cells; lane 2 was lysate of recombinant bacteria induced at 16◦C for 18 h; lane 3 was supernatant of *E. coli*-pGEX-4T-1-Cas12a lysate induced at 16 for 18 h; lane 4 was precipitation of *E. coli*-pGEX-4T-1-Cas12a lysate induced at 16◦C for 18 h. **(b)** Sodium dodecyl sulfate–polyacrylamide gel electrophoresis analysis of purified pGEX-4T-1-Cas12a protein. Lane 1 was lysate of the unpurified recombinant bacteria cells, lane 2 was lysate of the remaining protein solution after binding to GST packing, and lane 3-6 were lysates of the eluted purified pGEX-4T-1-Cas12a protein.


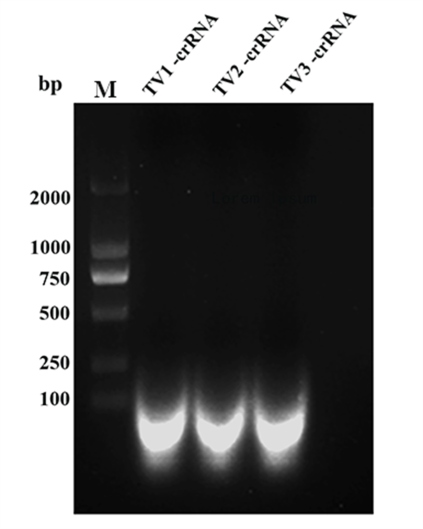


**Fig. S2.** Agarose gel electrophoresis of three purified crRNA nucleic acids.


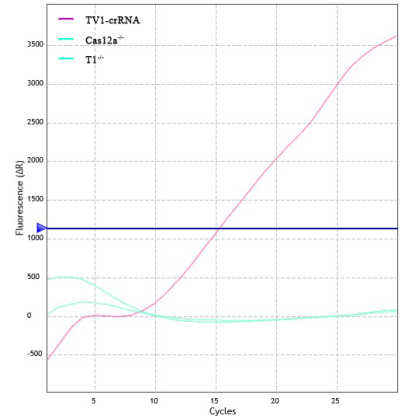

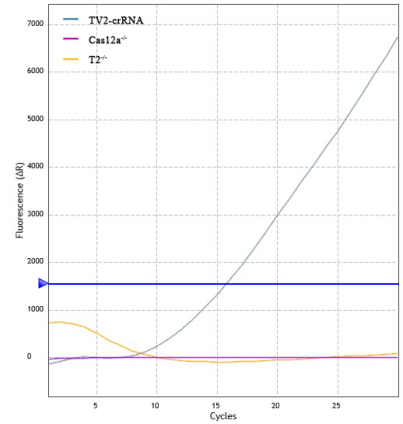

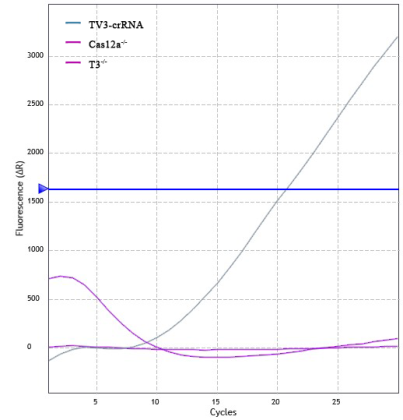


**Fig. S3.** Screening of crRNA for *T. vaginalis* detection by CRISPR-Cas12a. Cas12a^-/-^ and T2^-/-^ were negative control. Figure a, b, c showed the TV1-crRNA, TV2-crRNA, and TV/3-crRNA experimental groups, respectively.


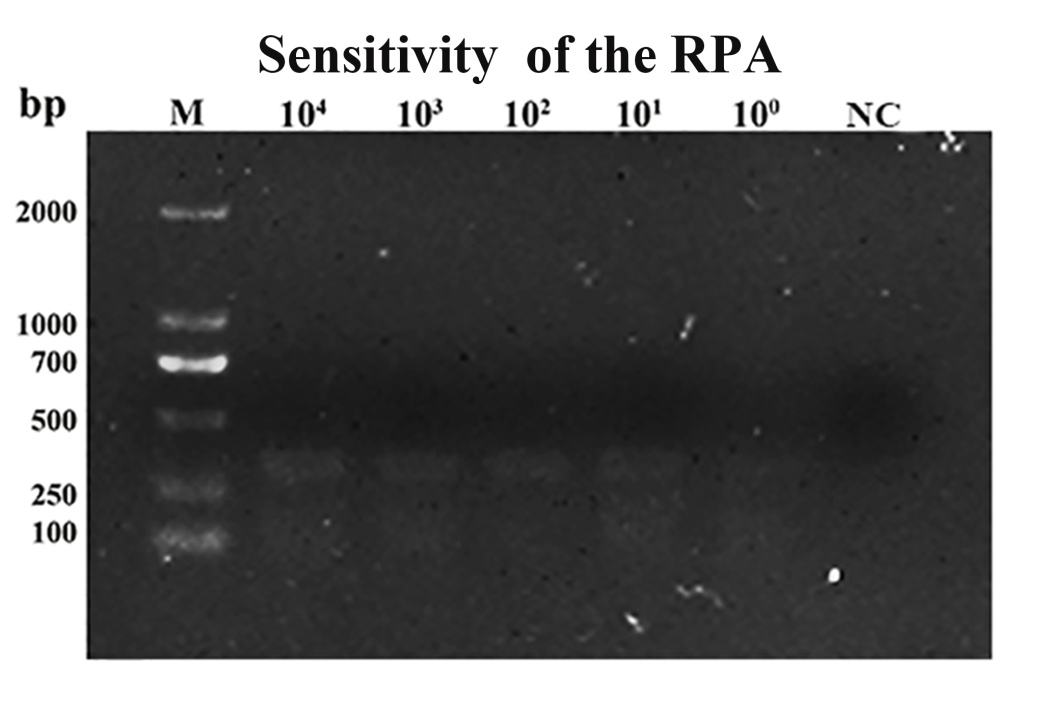


**Fig. S4.** Agarose gel electrophoresis of the pGEX-4T-1-*actin* positive plasmid after amplification by RPA at fold dilution to 1 copy/uL, with NC as a negative control.


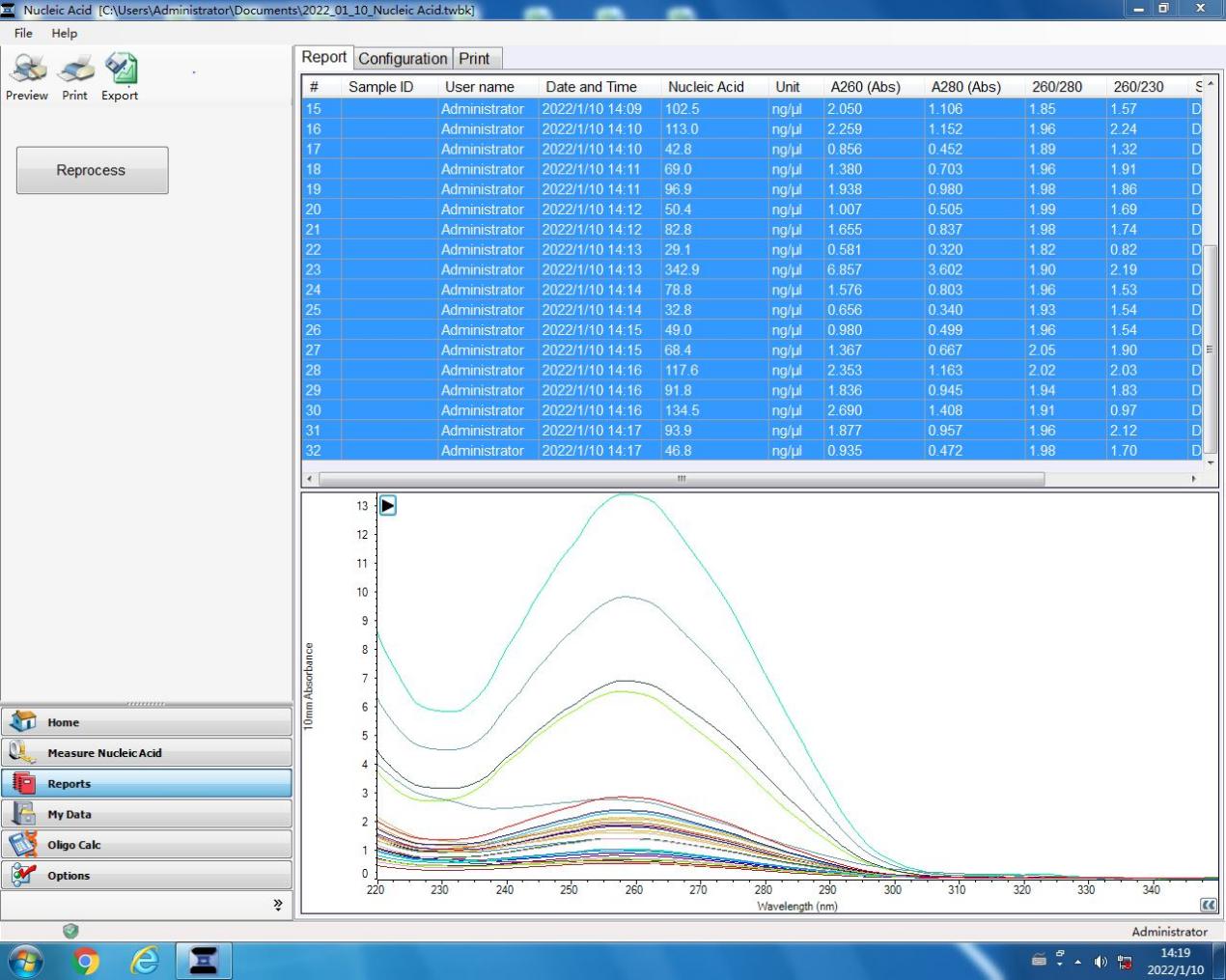


**Fig. S5.** Absorbance values of 30 clinical samples by Nanodrop.


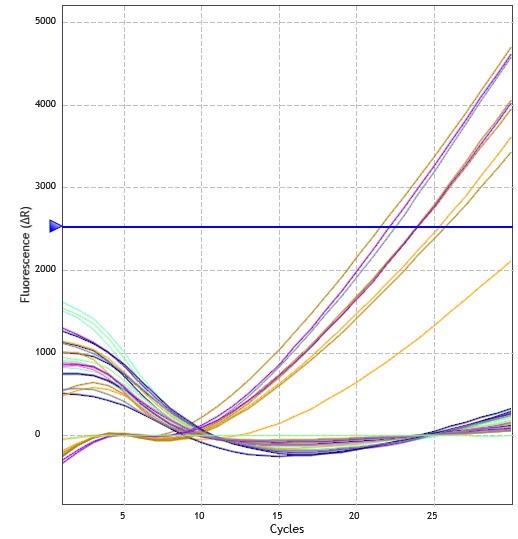


**Fig. S6.** Clinical samples detection by RPA-CRISPR-Cas12a.


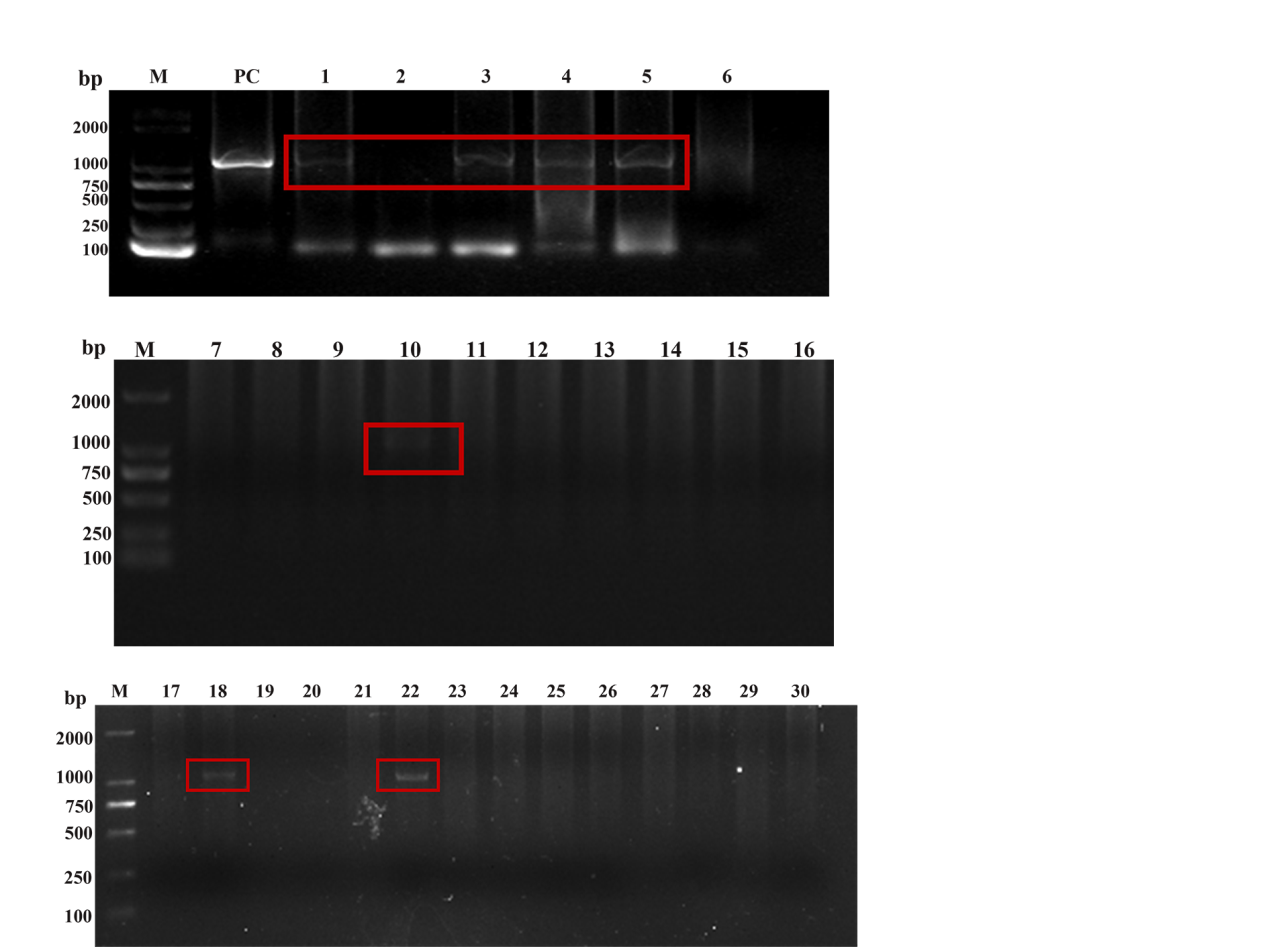


**Fig. S7.** Agarose gel electrophoresis of 30 human vaginal secretions by nested PCR.


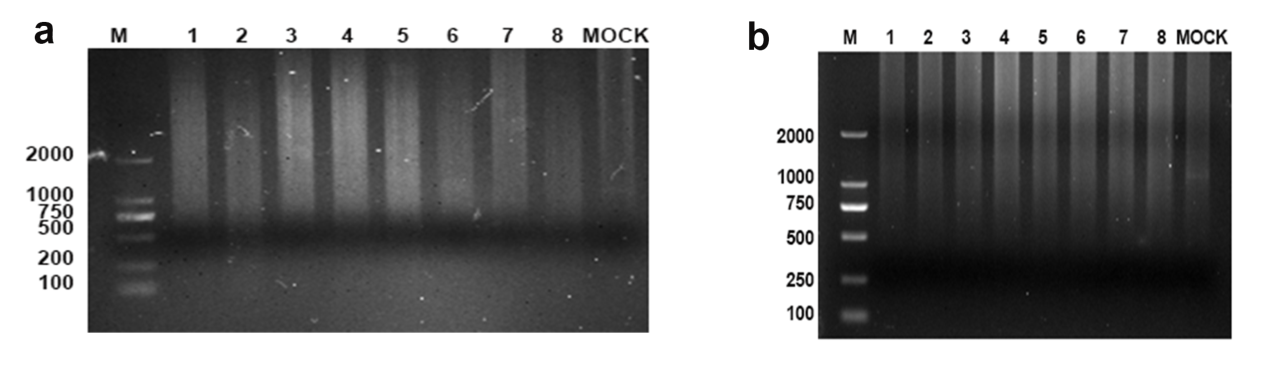


**Fig. S8.** (a) Agarose gel electrophoresis was performed on 9 male urine samples by nested PCR.(b) Agarose gel electrophoresis was performed by nested PCR on 9 semen samples.


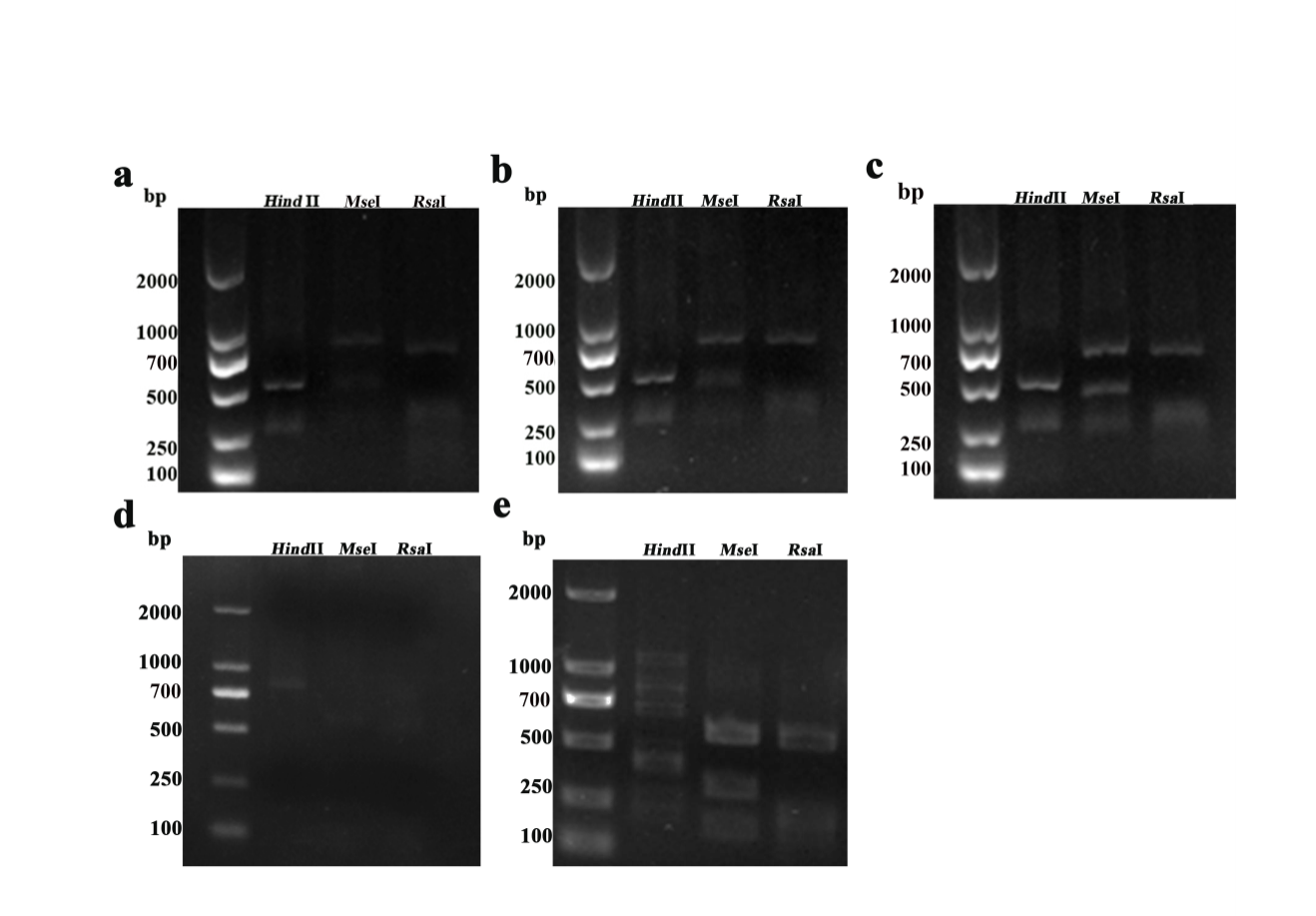


**Fig. S9.** Molecular typing of clinical positive samples based on the actin gene. a, b and c were Agarose gel electrophoresis of samples 1, 3 and 5, respectively.


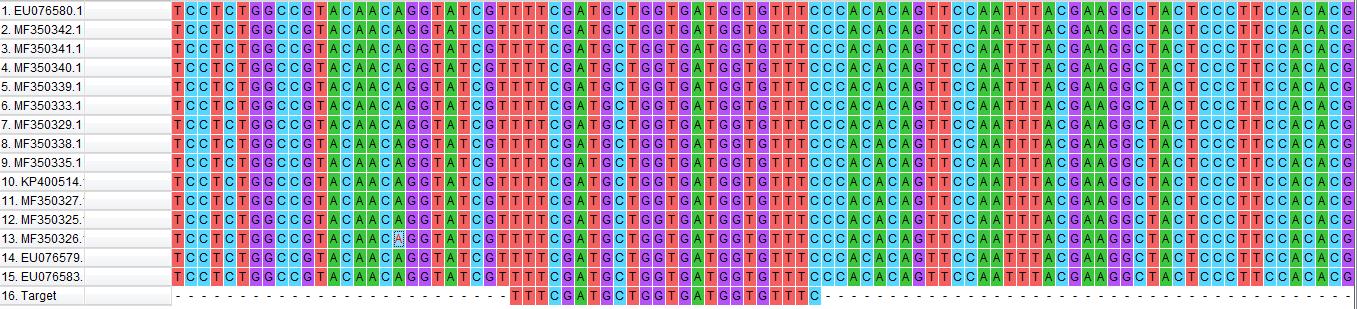


**Fig. S10.** Sequence alignment of target gene with different genotypes of *T. vaginalis* *actin* gene.
